# Limb salvage and systemic management of gouty tophi: Case series

**DOI:** 10.1097/MD.0000000000038137

**Published:** 2024-05-17

**Authors:** Xiaoyan Jiang, Anxin Li, Wei Hao, Cheng Yang, Hongyan Wang, Wuquan Deng

**Affiliations:** a Department of Endocrinology and Metabolism, Diabetic Foot Center, Chongqing Emergency Medical Center, Chongqing University Central Hospital, Chongqing, China.

**Keywords:** chronic refractory gout, diabetic foot ulcer, gouty tophi, limb salvage, negative pressure wound therapy, systemic therapy, wound healing

## Abstract

**Introduction:**

Gout is a chronic disease characterized by deposition of monosodium urate crystals. Tophi develop in some individuals with untreated or uncontrolled gout, which leads to ulcerations, cosmetic problems, mechanical obstruction of joint movement, joint damage and musculoskeletal disability. Currently, the treatment of gouty tophi is controversial and challenging. Both surgical and internal medical treatments have limitations and require further exploration in clinical practice.

**Patient concerns:**

In Case 1, we treated a patient with severe infection of diabetic foot ulcers with concomitant multiple gouty tophi in the same limb. A systematic management strategy was formulated to close the wound and save the limb. The ulcers healed successfully after half a year. In Case 2, a giant gouty tophi located in the first metatarsophalangeal joint of the left foot was removed by surgical treatment and vancomycin-loaded bone cement implantation. In Case 3, we present a case of gouty tophi that was resolved by standardized systemic medical management.

**Diagnosis:**

Three patients were all diagnosed with gout accompanied by gouty deposition, although there were other different comorbidities.

**Interventions:**

In case 1, we used debridement to gradually remove gouty tophi. In case 2, the giant gouty tophi was removed by surgical operation. In case 3, the gouty tophi disappeared after standardized treatment with medicine, diet and lifestyle management.

**Outcomes:**

Three patients underwent different treatment therapies to remove gouty tophi based on their specific conditions.

**Lessons:**

We explored effective interventions for tophi in gout by surgical or other interventions in combination with pharmacotherapy.

## 1. Introduction

The prevalence and incidence of gout and hyperuricemia have both increased in various countries over the last few decades.^[1]^ The pathogenesis of gout is well established, with a major role played by the deposition of monosodium urate (MSU) in joints and various other tissues such as subcutaneous tissues, and cartilage. Tophi are complex masses comprising MSU crystals surrounded by chronic granulomatous inflammatory tissue, which leads to bursal or peripheral joint swelling, tenderness, and pain. Tophi can develop owing to long-term hyperuricemia, which commonly develops in patients with chronic gout. Untreated or uncontrolled gout with tophi formation progressed to potential destruction, deformities, and dyskinesia of the peripheral joints.^[2]^

Diabetic foot ulcer (DFU) is a serious and full-thickness wound of the diabetic foot comprising a group of lesions, including vasculopathy, neuropathy, tissue damage and infection. Treatment of DFU remains challenging because of its high amputation rate and high mortality risk. DFU and gouty tophi, serious complications of 2 different metabolic diseases, usually exist alone but are rarely seen concomitantly in the same limb of a patient. Once the coexistence of DFU and gouty tophi in 1 foot impairs wound healing, the treatment becomes more complicated. Herein, we present a case of DFU with concomitant chronic multiple gouty tophi. Serious infection with DFU once threatened the patient life. A systematic management strategy was formulated to close the wound and save the limb.

On the other hand, although some systematic reviews have assessed the benefits and harms of non-surgical and surgical treatments for the management of tophi in gout, high-quality evidence is rare and requires further exploration.^[3]^ In this article, we describe the treatment methods of tophi by surgical or other interventions in combination with pharmacotherapy for common clinical cases and have achieved satisfactory treatment results.

## 2. Clinical cases

### 2.1. Case 1

Limb salvage and systemic management of DFUs with concomitant multiple gouty tophi.

A 53-year-old male admitted to the emergency department of our hospital because of left foot ulceration 14 days prior to presentation with a high fever for 1 day. He had an 8-year medical history of type 2 diabetes mellitus and > 10 years of gout and hypertension. Neither diabetes nor gout, the patients were not managed formally, and many complications gradually emerged. He was diagnosed with diabetic foot due to a left foot ulcer 1 year ago. The wound healed after treatment at the local hospital. However, his left foot suffered from ulceration again due to wearing inappropriate shoes 14 days prior to presentation. Therefore, he went to the local hospital to undergo routine blood tests, which suggested that the infection had increased. Radiography showed that the soft tissue of the left dorsum of the foot was swollen with air accumulation, and the bone of the middle phalange of the left fifth toe was absorbed, which was caused by infection. After debridement and dressing change, anti-infection and hypoglycemic treatment, his symptoms were not alleviated. There were multiple ulcers on the left plantar region, dorsum, and between the toes, with a large amount of purulent secretion spilling. He had a high fever of 39.3°C and was transferred to the emergency department of our hospital 1 day ago. He was diagnosed with diabetic foot infection and admitted to our department.

**Figure 1.**
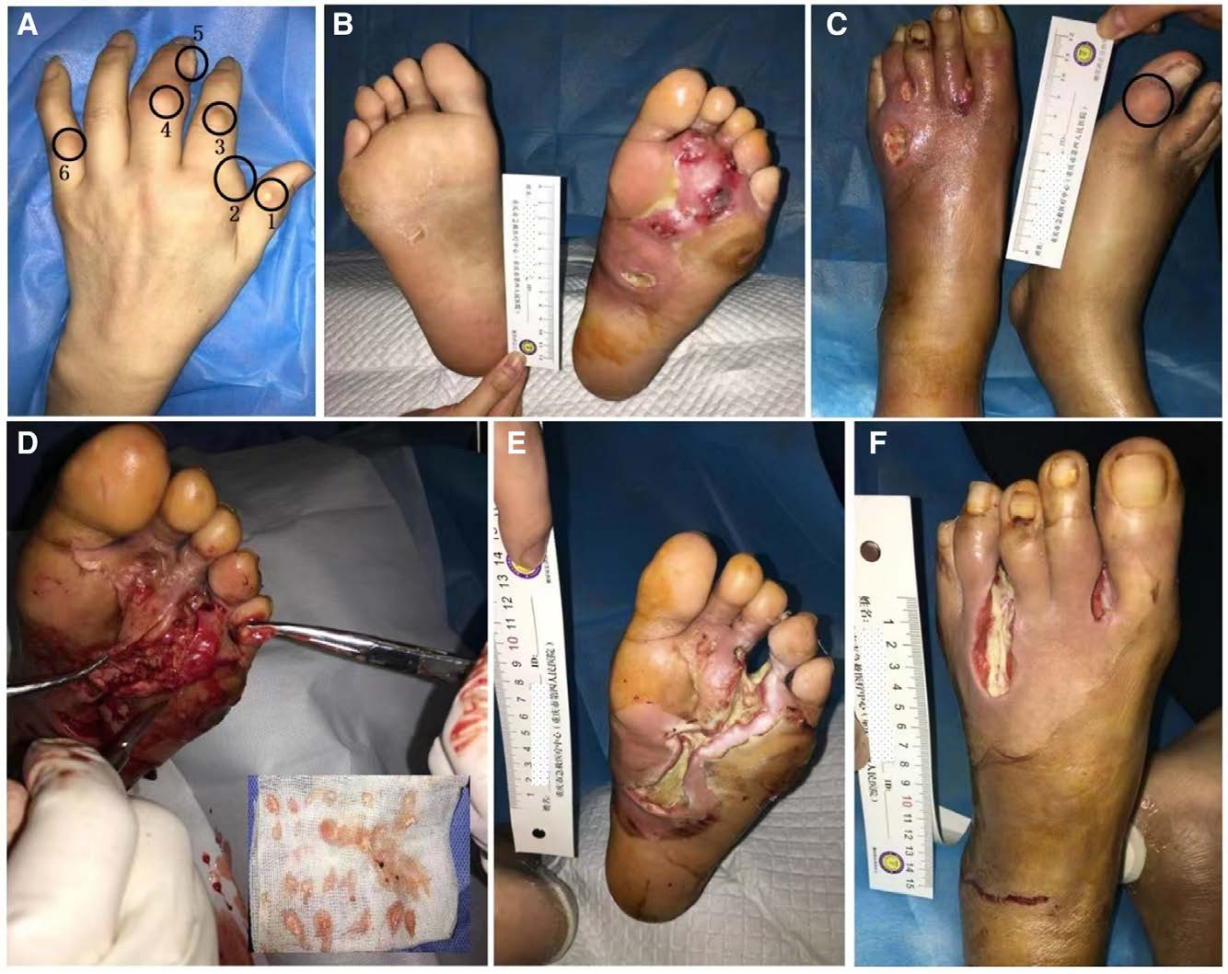
Gout tophi in the multiple interphalangeal joints and wound status in the left diabetic foot ulcers. (A) Gout tophi deposit in multiple interphalangeal joints of the left hand; (B) plantar appearance of both feet at admission; (C) dorsal appearance of both feet (left foot ulcers, gout tophi of the first metatarsophalangeal joint of the right foot); (D) a large number of gout tophi deposited in plantar tendons after sharp debridement; (E) and (F) show the appearances of the left plantar and dorsum 5 d after debridement.

**Figure 2.**
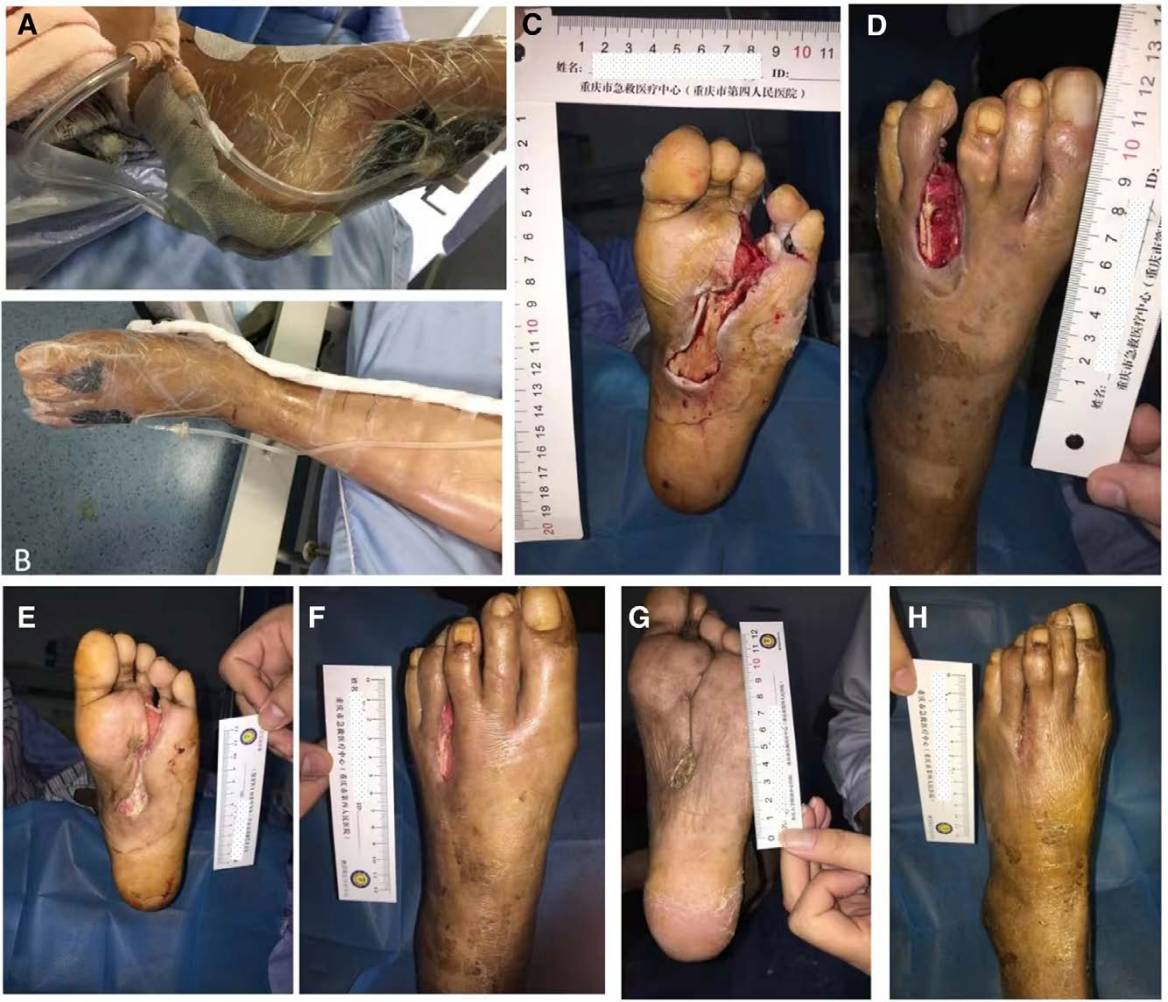
Treatment and healing of diabetes wounds. (A) Left foot negative pressure suction plantar; (B) The negative pressure of the left foot attracts the dorsum of the foot and between the toes; (C) The plantar wound was taken after 7 d of NPWT; (D) The wound of the dorsum after taking NPWT 7 d; (E and F) After debridement of 2 months, the plantar and dorsum of foot; (G) Wound healing of plantar; (H) Wound healing at the back of the foot and between the toes. NPWT = negative pressure wound therapy.

Physical examination revealed many gouty tophi of different sizes and shapes at the interphalangeal joints of both hands, as indicated by the black circular markings in Figure 1A and C. In addition, the gouty tophi deposition fluid flowed from the left heel (Fig. 2A). The joints were deformed, with some redness and swelling, a slightly elevated skin temperature, and no obvious tenderness. Multiple ulcers of varying sizes were observed on the left sole, dorsum, interdigital, and root of the foot, accompanied by a large amount of purulent secretions. The skin around the ulcer was significantly reddened and swollen, with necrosis and degeneration of the left sole skin, exudation of purulent secretions, and no obvious foul odor (Fig. 1B and C). The upper third of the left lower leg was swollen and reddened, with a significant increase in skin temperature. The dorsal arteries of both feet pulsate.

Laboratory investigations showed a random blood glucose of 6.38 mmol/L and hemoglobin A1c level of 12.7 %. The blood count and heart failure indicators were significantly elevated. The C-reactive protein level was 159.3 mg/L. Routine blood results showed that WBC 12.63 G/L and N count 10.45 G/L. N% was 82.7%. RBC 2.1T/L, and hemoglobin level was only 63 g/L. The Pro-*B*-type Natriuretic Peptide was 1266 pg/mL. Liver function tests showed an albumin level of 21 g/L. The patient serun uric acid was 360 µmol/L. T: 38.5°C, B: 126, R: 27, BP: 97/64 mm Hg. The patient was diagnosed with acute left heart failure, left diabetic foot infection, septic shock, acute gouty arthritis, and multiple gouty tophi. The patient was transferred to the intensive care unit because of progressive worsening of the condition, including sitting breathing with obvious breathing difficulties. After the patient symptoms improved, he returned to our department for further treatment 2 days later.

**Figure 3.**
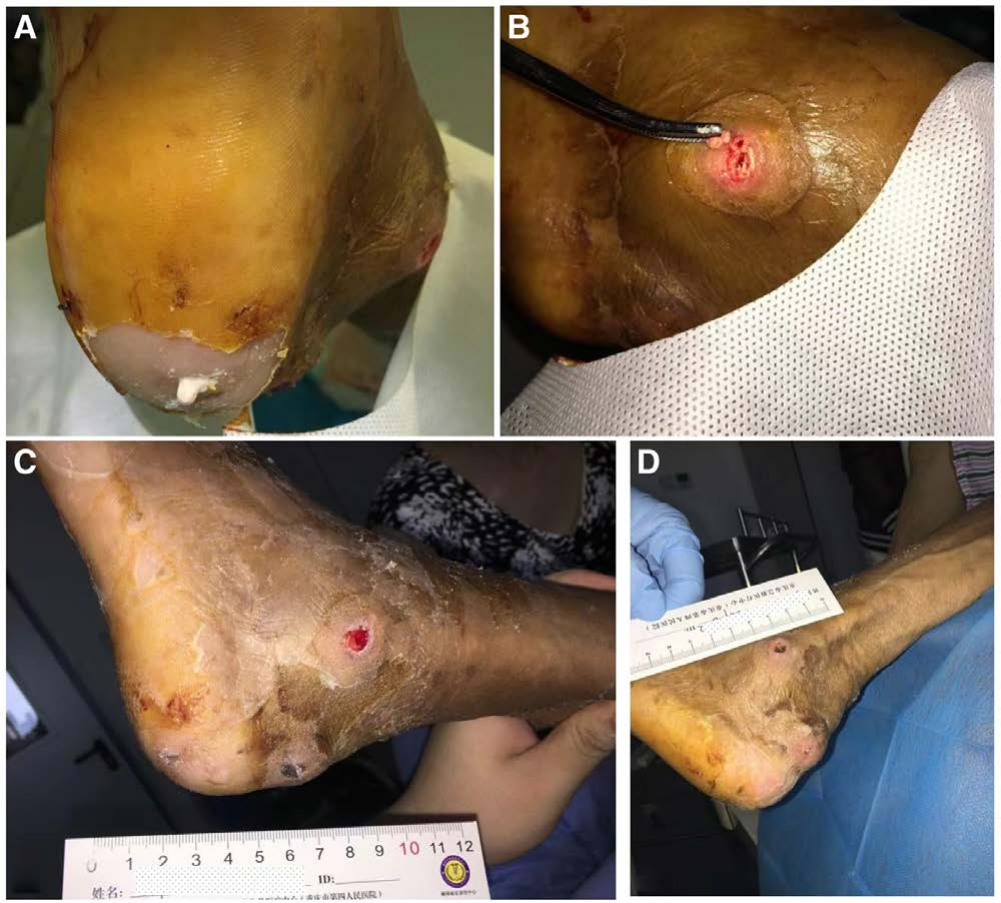
Gouty tophi in left foot. (A) The left heel gouty tophi overflowed at the time of admission; (B) Left lateral ankle gouty tophi debridement; (C) local debridement 7 d later, the heel and lateral ankle; (D) Heel and lateral ankle wound healed.

Based on the University of Texas (UT) Diabetic Foot Ulcer Classification System, the classification of the wound on the patient left foot was UT IV B. According to the International Working Group on the Diabetic Foot classification guidelines for diabetic foot wounds, the diabetic foot infection perfusion, extent, depth, infection, and sensation grade was 5. A series of standard medical treatments including antibiotics, were administered, blood glucose control was optimized, and peripheral circulation improved. In addition, the wound was thoroughly debrided, including wound cleaning and removal of all infected and non-viable (necrotic or dead) tissue. Meanwhile, a large number of gout tophi were deposited in the plantar tendons after sharp debridement (Fig. 1D). (Fig. 1E) and (Fig. 1F) showed the appearance of the left plantar and dorsum after 5 days of debridement. The wound was clean, and there was no purulent discharge. Negative pressure wound therapy (NPWT) with intermittent pressure (−50 to −150 mm Hg) was initiated to promote wound healing in the left foot (Fig. 3A and B). After 7 days, the negative pressure device was removed, and the wound color became rosy, with newly formed granulation tissue visible in the ulcer cavity (Fig. 3C and D). The wound significantly improved 2 months later (Fig. 3E and F). A small amount of gouty tophi was also observed in the external ankle wound (Fig. 2B). After removal of the gout tophy, the wound healed slowly (Fig. 2C). Complete wound closure was achieved nearly 6-months (Figs. 2G, H and 3D).

### 2.2. Case 2

Surgical removal of massive gouty tophi using vancomycin-loaded bone cement implants.

**Figure 4.**
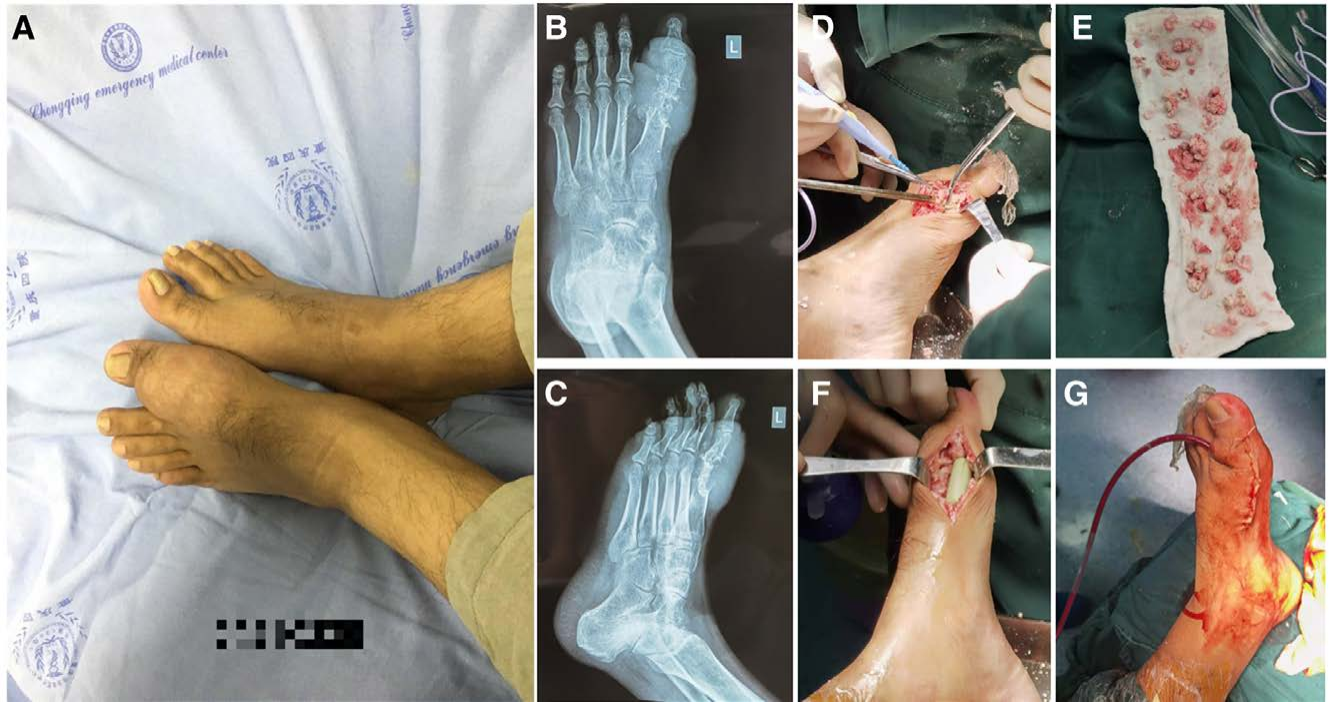
Surgical removal of giant gouty tophi in the first metatarsophalangeal joint of the left foot with vancomycin-loaded bone cement implant. (A) Appearance of giant gouty tophi of the left foot; (B) and (C) X-ray showed gouty tophi masses and bone defects in the first metatarsophalangeal joint in different positions; (D) After resection of non-viable bone and application of the vancomycin-loaded bone cement implant; (E) Massive removal of gouty tophi; (F) After resection of non-viable bone and application of the vancomycin-loaded bone cement implant (G) Wound suture combined with drainage.

A 26-year-old male was transferred to our department because of recurrent pain in multiple joints of the limbs for 5 years, which had worsened for 1 week. The patient presented with swelling and pain in the first metatarsophalangeal joint of his left foot 5 years ago without an obvious cause. He was diagnosed with acute gouty arthritis at a local hospital, and the symptoms improved after treatment. However, without proper treatment, gout has recurred several times in recent years. As the disease progressed, pain appeared in the first metatarsophalangeal, ankle, knee, and palmar and phalangeal joints of both feet. One week prior, there was significant swelling and pain in the first metatarsophalangeal joint of the left foot. After receiving treatment at the local hospital, the symptoms improved slightly, but due to the huge gouty tophi in the first metatarsophalangeal joint of the left foot, which affected to wear shoes and walk, he was referred to our hospital (Fig. 4A). Radiography showed gouty tophi masses and bone defects in the first metatarsophalangeal joint (Fig. 4B and C). Gouty tophi removal surgery was performed after multidisciplinary consultations and the evaluation of surgical indications and contraindications. Massive gouty tophi were removed from the affected area (Fig. 4D and E). The infected dead bone was resected and the space was filled with antibiotic-loaded bone cement (Fig. 4F). Antibiotic-loaded bone cement was prepared by mixing 10 g of Palacos G Bone Cement powder (PALACOS® R + G, Heraeus Medical GmbH, Germany) and 0.4 g. Finally, the wound was sutured with drainage (Fig. 4G).

### 2.3. Case 3

Gouty tophi resolution by standardized systemic management.

**Figure 5.**
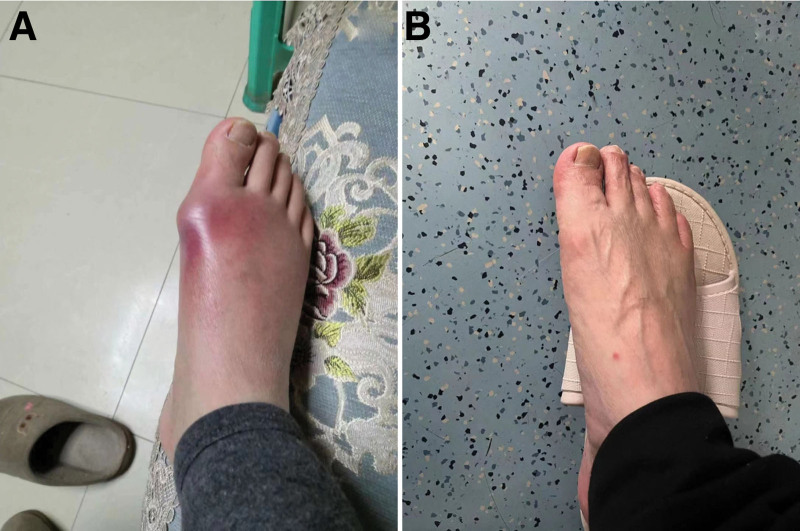
(A) Redness and swelling of the first metatarsophalangeal joint of the right foot with visible deposition of gouty tophi; (B) Gouty tophi resolution after 1 yr of medical treatment.

A 68-year-old male admitted to our department with redness, swelling, and pain in the first metatarsophalangeal joint of his right foot (Fig. 5A). Foot MRI showed massive gouty tophi deposition in the first metatarsophalangeal joint of his right foot. He was diagnosed with acute gouty arthritis with gout tophi deposition. He had a history of type 2 diabetes and had been taking oral drugs to control blood sugar for more than 9 years. He was discharged after receiving treatment for pain relief and uric acid lowering. After discharge, we conducted long-term standardized management and treatment, regularly visiting outpatient clinics every month to check the blood sugar and uric acid levels. One year later, the left first metatarsophalangeal joint gout tophi had dissolved and disappeared (Fig. 5B).

## 3. Discussion

To our knowledge, Case 1 is the first report of both DFU and gouty tophi occurring on the same limb. Initially, the patient general condition deteriorated rapidly, with severe infection and acute heart failure, which was life-threatening. The difficulty of treatment is obvious. Therapeutic strategies in the early stages of the disease are to save life, and then to save limbs. It is crucial to promptly evaluate and manage wounds when the patient general condition improves. Because of the complications of severe infection with DFU and gouty tophi deposition, wound healing is difficult and time-consuming. The treatment of diabetic foot combined with gouty tophi emphasizes the principles of comprehensiveness and individuality, saving the patients’ lives is the footstone of treatment, followed by avoiding amputation. The standard practices in DFU with tophi management include surgical debridement, dressings, wound off-loading, vascular assessment, infection control, pain relief, hyperglycemia and hyperuricemia long-term control, and multidisciplinary care.^[4,5]^ The treatment of DFU lesions combined with tophi may be because DFUs emphasize thorough debridement, while the removal of tophi may stop at the right time. Foot involvement is common in gout, with involvement of the first metatarsophalangeal joint and Achilles tendon.^[6]^ The tophi in this patient were located in the plantar and ankle joints. After exploring the wound, a large amount of tophi was discharged from the plantar wound. The other was located in the ankle joint, making treatment more difficult. Surgical treatments should be considered when impending or severe complications, including joint deformities that affect daily life, joint dysfunction with or without entrapping nerve, skin ulceration, sinus with or without infection, chalk-like material exudation, and tophi, are too large to be absorbed by themselves.^[7,8]^

Gouty tophi is a common and serious complication of gout that can lead to bony destruction, joint deformity and dysfunction, and even fracture.^[9]^ When diabetes or an infection is superimposed, the probability of this situation increases significantly. Glucocorticoid overuse, prolonged duration of tophi, and greater numbers of tophi are risk factors for ulceration in patients with gouty tophi.^[10]^ The purpose of surgical treatment is to improve function, relieve pain, improve appearance, remove sinus tract and reduce tophi deposition.^[11]^ In Case 2, we attempted surgical resection and bone cement implantation for a giant gouty tophi that severely affected function. Our previous study showed that vancomycin-loaded bone cement could be used as a common antibiotic and incorporated into a topical wound therapy system for the treatment of diabetic foot osteomyelitis.^[12]^

The systemic management of gouty tophi includes the following aspects: Diet and Lifestyle Management: Adjusting the diet by limiting the intake of high-purine foods, increasing water intake, and adopting a healthy lifestyle such as moderate exercise are beneficial for managing gout; Uric Acid Reduction Treatment: This involves the use of medications or other methods to decrease the levels of uric acid in the body, thereby preventing gout attacks and the deposition of gouty tophi. To dissolve the MSU crystals, reduce the number and volume of crystals, avoid the formation of new crystals, prevent recurrent gout flares, and reduce the serum uric acid concentration to <6 mg/dL by long-term urate-lowering therapy is effective and necessary.^[13,14]^ From our experience in Case 3, we reduced the serum uric acid concentration to <5 mg/dL in order to dissolve gouty tophi deposition. The comprehensive treatment goal of diabetes with multiple gout tophi is to reach the long-term standard of blood sugar and uric acid levels to ensure that they do not recur.

## 4. Conclusion

Management of gouty tophi in individuals with diabetes remains a challenge because of the lack of high-quality evidence for the management of tophi and the need for further studies. Adopting appropriate treatment strategies based on the different situations of patients is an important issue that physicians need to continuously explore in clinical practice. We hope that our treatment experience will provide a reference. More efforts should be made to explore effective interventions for gouty tophi in patients with diabetes.

## Author contributions

**Conceptualization:** Xiaoyan Jiang, Wei Hao.

**Project administration:** Xiaoyan Jiang, Cheng Yang, Hongyan Wang, Wuquan Deng.

**Writing – original draft:** Xiaoyan Jiang, Anxin Li.

**Writing – review & editing:** Xiaoyan Jiang.

## References

[1] Roddy E Zhang W Doherty M. The changing epidemiology of gout. Nat Clin Pract Rheumatol. 2007;3:443–9. 17664951 10.1038/ncprheum0556

[2] Wu Z Liu C Dai K. Intraspinal extradural gout tophus in the lumbar vertebral canal: case reports. Medicine (Baltimore). 2022;101:e28418. 35029886 10.1097/MD.0000000000028418 PMC8735772

[3] Sriranganathan MK Vinik O Falzon L. Interventions for tophi in gout: a Cochrane systematic literature review. J Rheumatol Suppl. 2014;92:63–9. 25180130 10.3899/jrheum.140464

[4] Everett E Mathioudakis N. Update on management of diabetic foot ulcers. Ann N Y Acad Sci. 2018;1411:153–65. 29377202 10.1111/nyas.13569 PMC5793889

[5] Richette P Doherty M Pascual E. 2016 updated EULAR evidence-based recommendations for the management of gout. Ann Rheum Dis. 2017;76:29–42. 27457514 10.1136/annrheumdis-2016-209707

[6] Dalbeth N Petrie K House M. Illness perceptions in patients with gout and the relationship with progression of musculoskeletal disability. Arthritis Care Res. 2011;63:1605–12. 10.1002/acr.20570 22034122

[7] Kasper IR Juriga MD Giurini JM. Treatment of tophaceous gout: when medication is not enough. Semin Arthritis Rheum. 2016;45:669–74. 26947439 10.1016/j.semarthrit.2016.01.005

[8] Carcione J Bodofsky S LaMoreaux B. Beyond medical treatment: surgical treatment of gout. Curr Rheumatol Rep. 2020;23:1. 33236200 10.1007/s11926-020-00969-6

[9] Khanna PP Nuki G Bardin T. Tophi and frequent gout flares are associated with impairments to quality of life, productivity, and increased healthcare resource use: results from across–sectional survey. Health Qual Life Outcomes. 2012;10:117. 22999027 10.1186/1477-7525-10-117 PMC3499162

[10] Zhengping H Xiuqi L Yuqi L. Clinical characteristics and risk factors of ulceration over tophi in patients with gout. Int J Rheum Dis. 2019;22:1052–7. 31119888 10.1111/1756-185X.13581

[11] Fang ZH Waizy H. Current concepts in the treatment of gouty arthritis. Orthop Surg. 2013;5:6–12. 23420740 10.1111/os.12024 PMC6583449

[12] Jiang X Li N Yuan Y. Limb salavge and prevention of ulcer recurrence in a chronic refractory diabetic foot osteomuelitis. Diabetes Metab Syndr Obes. 2020;13:2289–96. 32636663 10.2147/DMSO.S254586 PMC7335304

[13] British Society for Rheumatology Standards, Audit and Guidelines Working Group. The British Society for rheumatology guideline for the management of gout. Rheumatology (Oxford). 2017;56:1056–9. 28549195 10.1093/rheumatology/kex150

[14] Kiltz U Smolen J Bardin T. Treat-to-target (T2T) recommendations for gout. Ann Rheum Dis. 2017;76:632–8. 27658678 10.1136/annrheumdis-2016-209467

